# Selections and Abstracts

**Published:** 1914-06

**Authors:** 


### SELECTIONS AND ABSTRACTS

### THE UNDERLYING FACTORS IN THE SPREAD OF TUBERCULOSIS.

Albert Philip Francine, A. M., M. D., Philadelphia.

There are two broad phases in the communicability and spread of tuberculosis, which in orderly procedure are best discussed separately, (a) the social and economic side of the problem, and (b) the more strictly medical side.

### THE SOCIAL AND ECONOMIC SIDE

In placing the responsibility for the spread of infection we must in the last analysis begin with the individual, rather than the institution. It is, theoretically, more the fault of the people than the fault of their surrondings. It is the fault of the people who make the homes. This great plague flourishes in the homes of the ignorant, amid poverty and squalor; but, as has been well said, it is not alone the buildings which make the slums, but the people who live in them. Of course, bad housing conditions play a profound role in the endemicity of tuberculosis, but even could we give every family an airy, clean house, so long as ignorance, carelessness, filth, dissipation and alcoholism existed, we would still have slums, infected houses and tuberculosis.

It is perfectly apparent that better housing conditions would be an enormous stimulus to better living and better home management; and therefore, while the first remedial step is education and uplifting among the poor and ignorant themselves, the second, corollary need, which is of equally pressing importance, is the improvement of physical conditions of living—of housing conditions. Next in importance in this relation comes the subject of conditions of labor or occupation; for, aside from faulty ways of living and bad places to live in, there are the many vile conditions of labor, which reduce the health and weaken the resistance of the individual, making him susceptible to superinfection or a fresh infection, and rendering him and his descendants less and less likely and able to bring into the world and rear healthy, robust children.

In mentioning conditions of labor, however, as an important element in the prevalence of tuberculosis, we must think clearly and understand that the many existing bad conditions are largely predisposing factors only, and that their true significance relates to the demoralization of the health, morals and social status of the individual, and to home conditions from inadequate wages. Because the morbidity from tuberculosis is high among a certain class of wage-earners, we must not jump to the conclusion that that kind of work specifically "causes" tuberculosis. What it really does is to reduce the general health and resistance of the individual. If such workers have the seed of tuberculosis in their bodies, as is often the case, undue fatigue, confinement and low wages give rise to the physical conditions necessary for the flourishing of the seed. There is no specificity of labor as a "cause" for tuberculosis. It is an enormous factor, but never a cause (eliminating infected workshops). It comes the nearest to being a cause where certain forms of dust are constantly inhaled. which act (in one way at least) by reducing the tone and resistance of the lungs themselves, and so predispose to the pulmonary localization and activity of already existing infection, or a fresh infection.

For ethical and hygienic reasons the common drinking-cup must go, but tuberculosis is the least likely of the infections (so far as adults are concerned) to be engendered in this way. The question whether or not this should be made to include the common communion-cup may be answered perhaps in the affirmative on account of the danger of certain infections, but so far as the spread of tuberculosis is concerned, this is a negligible factor.

It is thus recognized that the problem of tuberculosis is in a large sense a social and economic one. With such factors to contend with, having their roots deep down in the very basis of modern life and conditions, affecting millions of people, is it not right that we should at least pause to appreciate the vastness of our undertaking, and from its very vastness should we not draw resolve not only to pursue our aim indomitably, but to be patient in demanding immediate results, though it should be stated, in passing, that results are becoming strikingly apparent.

Thus let us neither forget nor be dismayed by the fact that our problem is to combat, one might almost say, the drift of the times, to raise through education, sanitary laws, medical hygiene and philanthropic effort the proletariat from their condition of dense ignorance and poverty, to enable them to secure suitable homes and teach them how to live in an manage them, and to improve the conditions of their labor, that there may be cleanliness and light, suitable care of the children, sufficient food and clothing, necessary hours of rest, provident and good habits and self-restraint, and available medical supervision. But having said this much, we have not said all, for we must perform the same Augean task for the tens of thousands of immigrants arriving hourly from Europe to swell the ranks of this great army of ignorance and poverty.

But there is one satisfaction to be taken in the very breadth and vastness of this phase of the problem, namely, that it is not merely tuberculosis work, but public health work in whatever form of activity this shows itself. Antituberculosis work and all forms of public health work in general bear an increasingly apparent interdependence. It is not alone strictly antituberculosis measures which may be confidently looked to for results, but all the associated movements for the public welfare in health, morals and conditions, which are essentially allies. This is now fully recognized, and with the well-organized tuberculosis crusade pointing the way, other movements have followed along similar lines and the crusade of enlightenment and prevention of disease is becoming more and more a unit in its methods, aims and interrelationships.

THE MEDICAL SIDE

It is necessary next to consider certain salient features of the disease itself bearing on the question of the spread of infection. Two great factors in its communicability stand out:

1. Infection takes place from close personal association or contract with open tuberculosis, as by living with a consumptive or from living in a room or house contaminated by a consumptive. The great source of the infection lies in the carelessness of the individual consumptive in contaminating his surroundings by spitting about or not properly disposing of his sputum, or by spray infection from coughing (equally dangerous) without guarding the mouth with a paper napkin. His towels, bedding, table utensils, etc., are also a source of infection. The period at which a consumptive is most dangerous covers, of course, the second and third stages of his disease when the lung is breaking down and the sputum contains large numbers of living tubercle bacilli.

2. The age at which infection is most likely to take place is childhood. It should be borne in mind that adults, particularly healthy adults, have a very considerable resistance or immunity to tuberculosis infection. Without discussing the question of the source of this relative immunity, it may be stated as a fact that it requires a prolonged exposure and implantation to give rise to pulmonary tuberculosis in an ordinarily healthy man or woman. In proportion as bad surroundings, faulty personal hygiene, bad conditions of labor, other infections, dissipation, etc., play a part in the life of the individual, by just so much is this natural or acquired immunity impaired or even broken. But the fact remains that the chance inhalation of tubercle bacilli, as they may float in the dust of the street, or in public meeting-places, or the occupancy for a night or two of an infected room or sleeping-berth, could hardly give rise to pulmonary tuberculosis in an adult.

This must not be misinterpreted as in any sense condoning infected dust or infected places. No inference can be drawn from this that spitting in public places, street-cars or on the sidewalks should be tolerated. On ethical grounds and because of the dangers of catarrhal infections, spitting should be (and is in many communities) a misdemeanor punishable by law. The danger of infection of children by such conditions is a very possible one; for the time at which this natural resistance is weakest is in childhood, and for this reason and because of intimate and prolonged contact with open tuberculosis in their homes, children furnish the great soil for implantation. For physiologic reasons which it would lead us too far afield to discuss, this infection during childhood does not as a rule develop into pulmonary tuberculosis at that time, but lies dormant in the lymphatic system or is latent until adult life, when it breaks forth or manifests itself in pulmonary localization. It is largely children infected by contact in their homes who furnish later the ever on-coming crop of consumptive.

Milk and meat from tuberculous animals also constitute a source of infection in childhood. Infection may also be hereditary, the direct transmission of the tubercle bacillus taking place from the mother to the fetus by placental circulation. This has been shown to occur in mothers with advanced pulmonary or miliary tuberculosis, but has recently been shown as also possible when the lesion in the mother is in the incipient stage or even latent or non-active at the time of the birth (Warthin).

During childhood and early adolescence this early tuberculous infection, glandular in type, may manifest itself not at all, or only in anemia, underdevelopment, general physical delicacy, etc. These children react to tuberculin and often have enlarged lymphatic glands, but there is no other way in the majority of instances of telling that they are tuberculous; but this is quite sufficient for the diagnosis, which is to be understood to mean that the moment a child reacts to tuberculin, it is conclusive evidence that it has been sensitized by the tubercle bacillus and that the moment a child is thus tuberculized, it becomes potentially at least a future case of pulmonary tuberculosis.

Not only does the general trend of expert opinion bear out this view in relation to time of infection, but there are also many clinical and pathologic statistical data to confirm it. The results of the tuberculin test—which, when properly performed and repeated, is recognized as being specific—in large groups of children in hospital practice, as reported by von Pirquet, Calmette, Hamburger, Frantz and others in widely different cities, warrants the statement that most children of the working class are sensitized by the time they reach 14 or 15 years of age. The same is true of post-mortem findings among the poorer classes. Evidence has been clearly accumulating as to the frequency with which the disease is found at necropsy; and with increased refinement in post mortem work the percentages are increasing significantly. Nageli reported definite signs of tuberculosis in 97 per cent. of all bodies examined consecutively. Hamburger in a large group of children reported post-mortem findings of 63 per cent. with tuberculosis lesions between the ages of 7 to 10 years and 95 per cent. in those between 11 and 14 years of age. Ghon's post-mortem statistics from St. Elizabeth's Hospital in Vienna show that by the end of the third year from 6 to 8 per cent. are infected, the percentages rapidly rising until by the fourteenth year the infection reaches 92 per cent. It goes without saying that by no means all these patients would have developed pulmonary tuberculosis clinically had they lived; indeed, this early infection is looked on by many as a source of immunity to subsequent reinfection, but such statistics furnish valuable evidence of the time when primary infection takes place.

There arises here an interesting paradox, for while we know that infection with tubercle bacilli is so largely universal among the poorer classes in our cities, yet we also know that the death rate from tuberculosis among urban populations has been steadily decreasing. To what is this due? Is it due to strictly anti-tuberculosis measures in association with preventive and curative measures against disease in general? Is it due to institutional care and improved conditions of living? Or is it due to some deeper cause, a development of increased resistance, specific immunity or allergy (von Pirquet) in urban populations themselves? Recent discussion has concerned itself very much with this phenomenon, and authoritative opinions differ.

Thus Newsholme points out that, while the death-rate from tuberculosis is nearly always greater in urban than in rural districts, the countries showing the most urbanization have secured the greatest reduction in and the lowest death-rate from tuberculosis. The conclusion that he draws from this fact is that the death-rate from tuberculosis has declined to the greatest extent in those countries in which the ratio of institutional to domestic relief has been highest. A consideration of all the facts, he says, justifies the conclusion that the substitution of institutional for domestic relief for the consumptive poor has been historically the main factor in the reduction of this death-rate, and in a later paper he repeats that had it not been for the steadily increasing extent of institutional treatment of the sick, and especially of the consumptive sick, which has characterized most of our great centers of population, we should have experienced, not the decline of tuberculosis which has occurred, but a great increase in its prevalence. With this view I am in entire accord.

On the other hand, a no less distinguished investigator than Karl Pearson has suggested that the selective process of many years of heavy mortality from tuberculosis has left us with a more immune and resistant population. He would have us change our views as to the value of hospitals and sanatoriums in the prevention to tuberculosis, and devote all our efforts to raising the resistance of the individual and the race. In brief, he denies the value of our general campaign, says that we are all tuberculized, and that the only efficient source of prevention lies in developing this communal or race immunity, which is largely a matter of environment and heredity. T. D. Lister goes so far along these lines as to say that were it for a moment to be granted that it is possible to control the spread of tuberculosis by preventive measures, then the service rendered to urban populations would be dangerous instead of beneficial, because the development of what he considers the only important factor, namely, increased communal resistance, would be ignored; and he further adds that in his opinion the adult death-rate from tuberculosis may be taken as a measure of the loss of resistance to latent tuberculosis acquired in childhood.

A phase of this latter view is seen in much recent literature based on the studies of Roemer, Hamburger, von Pirquet, Baldwin and others, which has been recently emphasized by the last-named investigator, who believes that a primary infection in childhood is of value as a protection against subsequent reinfection by the production of a specific allergy or changed condition. As an illustration of this view may be cited his belief that the development of a strong allergy is at least one reason why miliary tuberculosis is not more frequent during relapses and extensions of pulmonary tuberculosis, particularly since we know that the bacilli themselves are often free in the blood-stream. Applying this, he feels that most adult tuberculosis is not due to a new infection or reinfection from contact, because the allergy of a childhood infecion, so universally present, has protected against this, but that most adult tuberculosis is due to a lighting up of the disease from the spread of autogenous superinfection brought about by extraneous conditions of environment. Thus he argues that adults are very little endangered by close contact with open tuberculosis and not at all in ordinary association; childhood is the time of infection, youth the time of superinfection, and that from extension of the primary disease. With this view, except as it might imply a relaxation of any of our usual precautionary measures against the possibility of infection among adults by contact, I am largely in accord, as stated above. Such theories inevitably lead back to the fundamental principle of eugenics, which lie not strictly in the survival of the fittest, not strictly in an attempt to rear a race of men and women whose resistance is attuned to living and surviving in a germ-laden world and who are capable of withstanding an utterly vile and vicious environment, but rather in forming a pure, healthy and happy environment, in making the world a fit place for all to live in, and then in producing the best and ablest race both physically and mentally to inhabit that purified environment (Moore).

Certain phases of the views quoted above appear radical and perhaps unwise general teaching (see Pearson), but whatever the element of error or of truth—and the truth probably involves a middle course—these views have at least served the purpose of calling the attention of those interested in the crusade to this very important element of racial and communal resistance, whether natural or acquired by early infection, which we have perhaps been inclined to overlook and neglect, and point to the practical necessity for directing every effort by good food, rest and recreation, by airy schools, playgrounds, inspection and supervision, to raising this quality of resistance, particularly in childhood and early adult life.

It may be stated as a fact that tuberculosis is the most common infection of childhood, and as well pointed out by Philip, we must get rid of the artificial distinction between so-called medical and surgical tuberculosis. From the scientific point of view, the most slender seedling of tuberculosis is potentially significant. It is impossible to say which tuberculous seed will be cast off and which will mature. Inoculation may occur through the mucous membrane of the gastro-intestinal or respiratory tract, or the skin, and whether or not it will spread from the lymphatic system, which is the first site of this early infection, and develop into pulmonary tuberculosis later depends largely on the child's vitality and resisting powers through its living tissue cells. In other words, this quality of natural or acquired immunity may hold the infection dormant; may heal an active lesion in its incipiency or localize it in the glands or bones; or may give way with resulting memingeal, miliary or pulmonary involvement. The course of events which supervenes is dependent largely on extraneous circumstances, on the amount and character of the tuberculous infection, on the number and character of the acute infections to which the person is exposed; on his enforced environment and to a considerable extent on inherited qualities. Philip says:

"The problem can only be solved effectively by a better understanding of the physiologic needs of developing life and a corresponding renovation of the nurseries and schoolrooms of the nation. It is folly to dream of transferring all tuberculous children to preventoria or sanatoria. This plan is to plead ignorance of the essential needs of the problem. The home of the poor man must be made the nursery of healthy children and cease to be the breeding-ground of tubercle-tainted wastrels. Each recreated home is an effective preventorium against tuberculosis."

It will thus be seen that the problem of prevention, to be effective, even as limited to the medical aspect of the communicability of tuberculosis, must take into account not only the care and isolation of the consumptive himself, but also the care and development of the children who have already been infected or who may be exposed to infection, and probably the development of a specific racial immunity.

But when we view as essentially one, as we must do, the two broad phases of the problem which I have attempted to outline, namely the social and economic conditions, and the more strictly medical conditions responsible for the prevalence and spread of tuberculosis, the point previously emphasized is brought forcibly on us—how very apparent is the interdependence to-day of the tuberculosis campaign and all efforts looking to the common welfare! Let me repeat that it is not alone by the strictly antituberculosis campaign that we may confidently expect to control tuberculosis, but by all allied movements looking to improvement of the health, morals, or condition of the people. Folks says:

"If our task is the more difficult because it is bound up with every phase of modern civilization, it is equally true that every substantial advance in other lines assists our cause."

For instance, it has been pretty conclusively shown (Reinicke) that wherever the death-rate from typhoid fever is reduced, through the introduction of pure water, numerically by one, there is a simultaneous reduction in the general death-rate of from two to three. What is true in this case is equally or even more strikingly so in relation to other infections. Think of the vista of accomplishment which opens up before us, when we know, as we do know, that more than 80 per cent. of all deaths are due to preventable causes!

Thus it is apparent that the movements against infant mortality, venereal diseases, alcoholism, the infectious fevers, cancer, procreation of mental defectives, and the correlated campaigns for better housing conditions, better hours and conditions of labor, child-welfare work, etc., all these movements, public or private, of whatever scope and by whatever methods they proceed, are all working to a common end, the welfare of the race, and as such are prototypes and allies of each other and of the greatest of them all, the tuberculosis campaign. They are all campaigns of preventive medicine, based on scientific development and attempting largely by education to carry the message of health and right living into the homes and very hearts of all the people.—Journal A. M. A., March 7, 1914.

---

### SEROBACTERINS (SENSITIZED BACTERIAL VACCINES)

Serobacterin is the Mulford name for sensitized bacterial vaccines, a new and improved form of bacterin possessing certain important advantages over the ordinary product.

### HOW SEROBACTERINS ARE PREPARED

Let us take Typho-serobacterin as an example. The first step is the production of an immune typhoid serum. Goats are used for this purpose. The reason for not using horses is that there may be a possibility of making persons treated hypersensitive, so that subsequent doses of horse serum might produce a severe reaction. Sensitization of this kind may be regarded as a laboratory curiosity, but in order to be absolutely on the safe side we are employing goats exclusively for the production of serum to be used in the manufacture of Serobacterins, as goat serum will not sensitize to horse serum. The goats are injected at stated intervals with typhoid bacilli until laboratory tests show a large amount of typhoid antibodies present in the blood. The animals are then bled and the blood serum is separated from the blood clot. The latter is discharged, the serum alone being employed. The serum from he goat is really an antityphoid curative serum.* In other words, it is a serum containing antibodies for the typhoid bacillus. (By antibodies is meant simply those substances in the serum which destroy or prevent the growth of bacteria.)

It is the antibodies which constitute the part of immune serum used in the preparation of the Serobacterins. After procuring a proper serum the next procedure is to combine the antibodies it contains with killed typhoid bacilli. To accomplish this, the bacilli are mixed with a suitable quantity of the serum and the mixture allowed to stand at laboratory temperature, with frequent shaking, for 24 hours. During this time the antibodies attach themselves to the typhoid bacilli permanently. The bacteria together with the attached antibodies, are then removed from the serum by centrifugation. (The centrifuge is an apparatus in which tubes containing substances to be separated are whirled around with great speed, the heavier substances settling to the bottom of the tube. The typhoid bacilli being heavier than the serum, are thrown to the bottom and the serum is then drawn off.) To remove every trace of serum, salt solution is added and the centrifuging repeated several times. This is called "washing." We now have a mass of typhoid bacilli combined with the antibodies which they have extracted from the serum. These bacilli are said to be "sensitized" because when injected into the body they are more rapidly acted upon by the tissues, and immunity is quickly established (24 to 48 hours.) When the bacteria are not sensitized before injection the body must produce the necessary antibodies. This takes from 5 to 10 days. Serobacterins are made up into suspensions (that is, the bacteria are mixed with salt solution, in the regular way, just as ordinary bacterins), and are standardized by estimating the number of bacteria per c. c. They are then placed in syringes ready for administration. Careful complement fixation and animal tests are employed to insure that proper sensitization has taken place. Since the value of Serobacterins depends on thorough sensitization, and the complement fixation test proves the extent to which sensitization has taken place, this test is a vital part of the technique.

*Antityphoid Serum is not marketed, as it has not been possible to secure a serum sufficiently rich in antibodies to be of much curative value; it is, however, potent enough to produce a good Serobacterin.

### EXCEPTIONAL SKILL AND FACILITIES NECESSARY

Serobacterins represent a product which can be successfully prepared only in a laboratory especially equipped for the purpose. The procedure is complicated and cannot be carried out except by the highest type of laboratory experts. The difficulties surrounding the preparation of sensitized vaccines have up to the present time prohibited their production for general use. The H. K. Mulford Company, as in the past with other important developments in bacterin and serum therapy, is the first to overcome these difficulties, and to offer for the first time this superior preparation to the general practitioner. It is important at this time to caution physicians who wish to use sensitized bacterial vaccines in their practice to specify SEROBACTERINS MULFORD, as by doing so they will be certain of securing a reliable SENSITIZED BACTERIAL VACCINE.

### THEORY OF THE ACTION OF SEROBACTERINS.

Just why this combination of bacteria and antibodies constitutes the ideal bacterin has so far not been satisfactorily explained. Logically it would appear that the antibodies would neutralize the bacteria and the mixture would be inert. That the reverse is true, however, has been convincingly demonstrated by prominent authorities (see below.)

### WHO ORIGINATED SEROBACTERINS.

Credit for originating Serobacterins, or sensitized bacterial vaccines, belongs to A. Besredka, Professor of Bacteriology in the Pasteur Institute. His work has been verified by such prominent investigators as:

Dr. Elie Metchnikoff (Assistant Director of the Pasteur Institute), who together with Besredka, conducted experiments on chimpanzees with sensitized typhoid vaccine (living) and demonstrated that the animals thus treated could be fed large quantities of virulent typhoid bacilli without harm. Metchnikoff is famous for the discovery of phagocytosis, and its bearing on immunity, his work on intestinal putrefaction and the use of the Bulgarian Bacillus in its treatment, etc.

Dr. Theobald Smith, Professor of Comparative Pathology, Harvard Medical College, Director of Antitoxin and Vaccine Laboratory State Board of Health of Massachusetts, who showed that a mixture of diphtheria toxin and antitoxin, or, in other words, SENSITIZED DIPHTHERIA TOXIN constitutes an effective vaccine for the prevention of diphtheria.

Dr. Charles Dopter, Physician-in-Chief, and Assistant Professor at the Hospital Val de Grace, who has achieved prominence for his work on the serum treatment of cerebrospinal meningitis, dysentery, etc. His experiments with SENSITIZED DYSENTERY BACILLI demonstrated that sensitization converts even this extremely toxic organism into perfectly harmless vaccine, though up to the present time a dysentery vaccine of the ordinary variety (unsensitized) has not been used on account of the very toxic properties possessed by the dysentery bacillus even after it is killed.

Dr. A. Marie (of the Pasteur Institute) prominent for his work on rabies, tuberculosis and tetanus. He has been conducting extensive researches with SENSITIZED RABIES VIRUS. The results of these experiments led to the adoption of sensitized rabies vaccine instead of the regular Pasteur treatment on account of the more prompt action and the small number of doses required.

Dr. A. Calmette, Director of the Pasteur Institute at Lillve, together with an associate, Dr. F. Meyer, have demonstrated the absence of toxicity in SENSITIZED TUBERCULIN. They had very encouraging results with the use of tuberculin thus prepared in treatment. As a result of their investigations, sensitized tuberculins are now being quite generally employed in Germany. Dr. Calmette is well known as the originator of the ophthalmic tuberculin test, etc.

M. H. Gordon, Assistant Pathologist at St. Bartholomew's Hospital, London, who made important clinical investigations with sensitized STREPTOCOCCIC VACCINE or Streptoserobacterin in the treatment of erysipelas, etc.

The work of the above-mentioned scientists, as well as that of numerous other investigators (see Bull. No. 18) has verified beyond question the law laid down by Besredka that "WHATEVER THE NATURE OF THE VIRUS, WHETHER THE MICROBE OF PLAGUE, DYSENTERY, CHOLERA OR TYPHOID FEVER, OR WHETHER THE VIRUS OF RABIES, OR THE TOXIN OF DIPHTHERIA, WHETHER THE MICROBES ARE KILLED OR LIVING, SENSITIZATION CONFERS UPON THEM PROPERTIES WHICH CONVERT THEM INTO VACCINES OF THE FIRST ORDER, POSSESSING AN ACTION SURE, RAPID, INOFFENSIVE AND DURABLE."

### ADVANTAGES OF SEROBACTERINS.

1. RAPID ACTION.—The immunity secured by the use of Serobacterins appears so quickly that it is effective within 24 to 48 hours. The advantage of this prompt action is realized when it is considered that 5 to 10 days are required before the immunity following the first dose of the ordinary bacterin is present. (This of course applies to healthy persons when the bacterin is used for preventive purposes. In treatment the response following the injection of the ordinary bacterin is quicker than in immunization, but not as prompt as that following the use of Serobacterins. This will be explained in subsequent papers.)

2. ELIMINATION OF REACTIONS.—No "local reaction" (soreness at the place of injection) or "general reaction" (headache, chilliness, and feeling of illness) follow the injection of Serobacterins. These "reactions" attend the administration of the ordinary bacterins, varying in degree with different patients.

3. DURABILITY AND PERMANENCE.—Immunity secured by the use of Serobacterins is even more potent and lasting than that secured by the ordinary bacterin. This has been proven by animal experiments.

4. HARMLESSNESS.—Serobacterins are absolutely harmless, and may be used in treatment at any stage of the disease. Following the injection of the ordinary bacterin there is a slight lowering in the content of bacteria-destroying properties of the blood due to absorption of these protective substances by the bacterin. This is known as the "negative phase." It is generally considered of no clinical importance, but is entirely eliminated by the use of Serobacterins, which, being already combined with antibodies, do not absorb any of these present in the blood of the patient.

### HOW SEROBACTERINS ARE USED

SEROBACTERINS SHOULD BE USED BY PREFERENCE IN EVERY CASE WHERE BACTERIN TREATMENT OF ANY KIND IS INDICATED. On account of their harmlessness they may be used even in the very latest stages of a disease. In such cases it was heretofore thought that only serums could be of any value. The reason for this belief was that the patient could not any longer produce antibodies, and that it would be necessary to introduce them from without, which could be accomplished only by administration of curative serum. With the Serobacterins, however, it has been shown that even in these cases, good results frequently follow their administration. Serobacterins do not interfere with the administration of serum as a supplementary treatment in the usual manner.

---

### AUSCULATORY INFLATION OF THE COLON.

A New Method of Outlining the Position of the Colon.

By John H. Musser, Jr., B. S., M. D., Philadelphia.

The importance and necessity of outlining and determining the position of the colon in abdominal disorders is frequently brought to the physicians attention. This need, particularly important from a diagnostic standpoint, is readily apparent to those interested in gastroenterology as well as in general practice. Especially is this so since the much discussed colonic stasis has so frequently been considered the etiological factor in a myriad of vague disorders. If anatomical or pathological alterations in the colon can be definitely shown, there is at once evident a possible seat of present or future trouble.

The great difficulty in the past, however, has been to find a method by which the position of the colon could be definitely outliend. Two procedures have been employed, 1, the Rontgen ray, and 2, inflation of the colon with air. The objections to the first method are primarily the expense, and, secondarily, the time consuming and often anoying preparation for examination. These objections in many cases are invalid; some patients have the money and the time to give to this examination, while others, patients in the public wards, have the time, and the expense falls upon the hospital. In many patients, however, these are very real objections, and they apply particularly to the class of patients upon whom X-ray has been done, namely, the patients in the medical dispensary. Unless these patients are willing to go into the ward for one or two days, this type of examination must be foregone. When the wards are full, or when the patients cannot afford to give the time, even if they are willing to do so, a satisfactory Rontgen ray examination is out of the question. The same remarks apply to the great body of moderately well to do patients, who are able of pay reasonable fees, but cannot afford larger outlays, unless absolutely necessary. In these types of patients, resource must be had to inflation of the colon with air, a most unsatisfactory procedure in any case. Unless the patient has thin abdominal walls it is impossible accurately to see or percuss the position of the colon. To obviate this difficulty, I have been employing a method the past year and a half, which, as far as I know, has never been used before. It is an adaptation of the well known and extensively employed ausculatory inflation of the stomach and has, I believe, the same relative value up to a certain point.

The method I employ is as follows: The patient is instructed to have the bowels well emptied before coming to the dispensary. Usually a simple cathartic the night preceding the examination is sufficient, though at times I have advised them to take cleansing enemas immediately before coming to the dispensary. When ready for the examination, a thick, rather stiff rectal tube is put, by the patient or the nurse, as high into the rectum as possible with comfort to the patient. I use a tube that will project from the rectum about twenty to twenty-four inches so that it can be manipulated easly from any position and may be brought out from beneath a sheet or the clothes without exposure of the patient. A strong air bulb is then placed on the distal end of the tube. With the patient in the prone position and the abdomen uncovered, the stethoscope bell is placed upon the lowest portion of the abdomen. The bulb is then squeezed and after each squeeze the stethoscope bell is moved upward an inch or two. When the transverse colon is reached, the character of the inflation sound is distinctly changed, and from a rather muffled distant sound, becomes a much clearer, louder sound with a rather metallic tone, while the passage of the air can be distinctly heard. The same procedure is repeated in every direction and the first change of tone is marked on the skin with a dermographic pencil. In this way the entire outline of the colon is marked off on the abdomen. The patient can now be made to stand and the outline in the standign position may be obtained. There is usually on difficulty in securing a noticeable change in the inflation tone over the sigmoid flexure, the descending and transverse colon. The tone is not always clear, however, in the region of the hepatic flexure and the ascedning colon, but becomes very distinct and unmistakable, as a rule, over the cecum. With this method abnormal variations in the large intestine are readily appreciated. A redundant sigmoid, a ptosed colon, a dilated and movable cecum, and other abnormalities are usually clearly revealed.

I have been able to control seven of these inflations by fluoroscopic examinations, made at the University of Pennsylvania Hospital by Doctor Pancoast and Doctor Garves. Before the Rontgen ray procedure, I would diagram my findings and write down exactly what I found upon ausculatory inflation. I would then compare them with the reports and findings of the radiographers as well as what I saw during the fluoroscopy. In six of these cases the two findigns corresponded accurately. In the seventh they did not do so, but on repeating the ausculatory inflation test, my results corresponded with those of the Rontgen ray.

I believe in view of the foregoing uniformly correct findings by the ausculatory method, that it is a method of diagonsis which often may be substituted for Rontgen ray examinations and which will secure a correct diagnosis in those patients who are unable or unwilling to undergo the bismuth examination of the intestines.

During the coures of the rather numerous inflations I have done, I have had the opportunity of making other observations. For example, I have found Bastedo's sign of appendicitis very trustworthy and present very uniformly in cases diagnosed as chroinc appendicitis, though, so far, only two of these have been actually verified by operative procedure. In a very few cases, I have, I believe, been able to diagnose several times partial mechanical obstruction. This is accomplished, not by the ausculatory phenomena, but by the subjective sensations of the patient. After a certain amount of air has been blown itno the bowel, the normal patients ahve a feeling of moderate distress which is promptly relieved, upon removing the bulb, by the passage of air from the end of the tube. In the presence of a mechanical obstruction, the air is not promptly passed, but is retained for some time. With this there is sharp, colicky cramplike pains in the region of the suspected obstruction, due no doubt partially to the increased peristaliss, induced by the mechanical effect of the air. These cramplike pains are not relieved until a large part of the air is passed. In these patients it is possible often to obtain a history of mechanical obstruction entirely unsuspected before the inflation of the large bowel.

The information ascertained by inflation of the bowel is of such value that the proceudre should be practised extensively. Certainly, knowledge of vague intestinal disturbances can often be gained by inflation that will point to the true cause of the disorder without the necessity of expensive rontgenographic examinations.—New York Medical Journal.

---

In differentiating syphilitic from other bone lesions a negative Wassermann reaction is not diagnostic.

---

The error is often made by capable Roentgenologists of mistaking the normal bone grooves of meningeal arteries for lines of skull fracture. Familiarity with the location of these grooves and comparative radiographs of the opposite side will obviate such an error.—American Journal of Surgery.

